# Information-Seeking Behaviors of Medical Students: A Cross-Sectional Web-Based Survey

**DOI:** 10.2196/mededu.4267

**Published:** 2015-06-29

**Authors:** Aoife Marie O'Carroll, Erin Patricia Westby, Joseph Dooley, Kevin E Gordon

**Affiliations:** ^1^ Dalhousie University Halifax, NS Canada; ^2^ Division of Pediatric Neurology, Dalhousie University Halifax, NS Canada

**Keywords:** information-seeking behavior, information retrieval, Internet, medical education, medical students

## Abstract

**Background:**

Medical students face an information-rich environment in which retrieval and appraisal strategies are increasingly important.

**Objective:**

To describe medical students’ current pattern of health information resource use and characterize their experience of instruction on information search and appraisal.

**Methods:**

We conducted a cross-sectional web-based survey of students registered in the four-year MD Program at Dalhousie University (Halifax, Nova Scotia, and Saint John, New Brunswick, sites), Canada. We collected self-reported data on information-seeking behavior, instruction, and evaluation of resources in the context of their medical education. Data were analyzed using descriptive statistics.

**Results:**

Surveys were returned by 213 of 462 eligible students (46.1%). Most respondents (165/204, 80.9%) recalled receiving formal instruction regarding information searches, but this seldom included nontraditional tools such as Google (23/107, 11.1%), Wikipedia, or social media. In their daily practice, however, they reported heavy use of these tools, as well as EBM summaries. Accessibility, understandability, and overall usefulness were common features of highly used resources. Students identified challenges managing information and/or resource overload and source accessibility.

**Conclusions:**

Medical students receive instruction primarily on searching and assessing primary medical literature. In their daily practice, however, they rely heavily on nontraditional tools as well as EBM summaries. Attention to appropriate use and appraisal of nontraditional sources might enhance the current EBM curriculum.

## Introduction

The information landscape is expanding rapidly, in large part due to the advent and evolution of the Internet. In the developed world, widespread access to the Internet along with intuitive and user-friendly search tools have made information on a vast range of topics available within moments. Some tools have gained such ubiquity as to become part of common parlance: “Google” is now a dictionary-approved verb [1].

Medicine has seen a similar trend, and the volume of information is not unequivocally helpful to practice. Authors have previously cited the challenges of staying up to date on a multitude of articles [2] and guidelines [3]; today, even the tools to access evidence proliferate. Whereas evidence-based medicine (EBM) developed as an approach to manage the challenge of translating primary evidence into clinical practice, the field has evolved to define increasingly sophisticated approaches to the body of literature as a whole. Indeed, some authors suggest that information management training may be as important as instruction on searching the primary literature [4,5].

Instruction in EBM is variable, however, and whether it affects long-term behaviors is uncertain [6-8]. The realities of daily work can present barriers to evidence-based practice, which is often perceived as a time- and effort-intensive pursuit [8-10]. Moreover, access to primary medical literature, summaries, and clinical support tools often comes with costly subscription fees.

These challenges make the user-friendly, freely accessible tools that are useful for general purpose inquiries appealing. Indeed, studies of medical trainees and practicing physicians support the popularity of general purpose tools for clinical or academic queries [9,11,12]. Notwithstanding concerns regarding reliability, some evidence suggests that general resources such as Google can be effective in answering clinical questions [13-15].

If these issues are important for the future of medicine, we need to understand how information-seeking behaviors develop. Medical school lays foundations of knowledge and behavior patterns. Students’ active engagement in participatory knowledge building is critical to this process, and thus information acquisition and use have central importance. Students turn to information sources to build background knowledge and will subsequently develop increasingly patient-specific questions and searches. As clinical encounters lead them to continually integrate this knowledge, they gradually build mental maps that enable automatic processing for quick clinical decision making [16]. Meanwhile, however, they must recognize the ongoing need to engage with information sources: to update their mental maps and to supplement when prior knowledge is absent or insufficient [16]. Their medical education must therefore prepare them as managers of information as well as experts in human health.

Previous studies that focused on medical students in the developed world have considered the use of specific resources [17-19] or technology [20-22]. Others have reported on experimental educational interventions [23-25]. With the current study, we sought a holistic characterization of the information-seeking behaviors of a medical student cohort in the context of an existing formal EBM curriculum.

We surveyed students enrolled in a medical doctorate training program regarding their use of Internet resources for medical information and the instruction they have received in EBM and information management. We hypothesized that this group would report high use of general purpose resources with minimal instruction in the use or interpretation of such resources.

The primary objective of this study was to define students’ current patterns of resource use. We also sought to characterize student experiences of current informatics instruction. Lastly, we began an inquiry into student valuations of various resources for medical information.

## Methods

### Survey Instrument

We developed a web-based survey to assess student information-seeking behavior, formal instruction on information searches, and evaluation of sources of health information. The survey was developed based on a review of relevant published literature and pretested with a convenience sample of ten medical students. The final survey contained 20 questions, predominantly requiring yes/no or rating scale responses; one question required a numeric estimate, one asked students to list their five most-used resources, and two were open-ended. Questions regarding resource use were based on recall of the previous seven days. The list of survey questions is included in Multimedia Appendix 1.

### Participant Recruitment

All students registered in the Dalhousie University 4-year medical doctorate program, at either the Halifax or the Saint John site, were considered eligible for participation in the survey. At the time of the survey, there were 462 registered students. We considered students in years 1 and 2 preclinical and those in years 3 and 4 clinical because the first two years are classroom-based while the third and fourth years take place predominantly within clinical settings.

The survey was published online using the Opinio 6 survey platform (ObjectPlanet, Inc) and remained open for two weeks. Two of the study authors (AO and EW) made in-class announcements to each student cohort, and invitation and reminder emails were sent to eligible students.

### Data Analysis

The survey responses were exported from the survey platform into Excel (Microsoft Corp). Data were analyzed using descriptive statistics, computed by hand. Frequencies were reported as percentages. Where appropriate, 95% confidence intervals were calculated using the .cii command with exact binomial confidence intervals using STATA version 12.1 (StataCorp LP). No correction was made for multiple statistical testing.

One question asked respondents to list their five most-used resources from the previous seven days. We assessed response frequency, and used Wordle [26] to generate a graphical representation of this data in which type size reflects frequency of occurrence.

Selected data were subsequently graphed using R (The R Foundation). In keeping with a paper presented at the 2011 Joint Statistical Meeting [27], these Likert data were presented graphically, using diverging stacked bar charts across information sources.

Open-ended questions were analyzed qualitatively for themes by AO. Responses were read and common themes identified; on second reading, responses were categorized into thematic groups. Responses that reflected more than one theme were included in each relevant thematic group.

### Ethics

The study was formally reviewed and approved by the IWK Health Centre Research Ethics Board. We also received approval from the Dalhousie Undergraduate Medical Education Curriculum Committee.

## Results

### Participant Recruitment

Of 462 students invited to respond, 213 (46.1%) provided evaluable responses. Two-thirds of closed-ended questions received response rates of 42.0% or above (193 or more students), and all but one question had responses from at least 39.0% (180 students). Ten students accessed the survey but did not provide any responses; these were considered to be nonrespondents and were not included in the response rate figures.

### Study Participants

Of the respondents, 56.6% (120/212) were female and 42.0% (89/212) were male; 3 preferred not to answer this question. Preclinical and clinical training levels received comparable representation (110/212, 51.9%, and 102/212, 48.1%, respectively). Roughly half (109/207, 52.7%) had prior experience as contributors to peer-reviewed literature, while relatively few (35/207, 16.9%) had posted information online for the public.

### Instruction on Information Searching

While 80.9% (165/204; 95% CI 74.8%-86%) of respondents recalled receiving formal instruction on searching for health information, 67.1% (139/207; 95% CI 60.3%-73.5%) also recalled being discouraged from using certain resources. Education regarding bibliographic databases (eg, PubMed) was common (162/206, 78.6%; 95% CI 72.4%-84.0%), whereas respondents seldom had instruction regarding general purpose Internet sources. Only 11.1% (23/207; 95% CI 7.2%-16.2%) reported teaching regarding the general search engine Google, and fewer had education on Wikipedia (a free, online, open-content encyclopedia) or social media. Although self-rated competence in finding information showed some variability, a strong majority (184/207, 88.9%; 95% CI 83.8%-92.8%) felt their skills were good or better.

### Pattern of Resource Use

**Figure 1:**
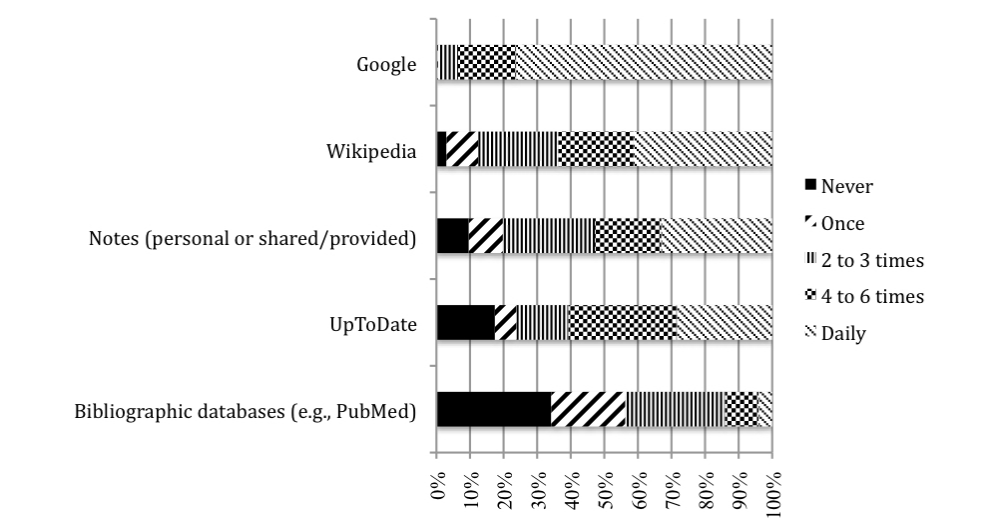
Student self-reported use of resources in the previous 7 days.

We considered a resource to be heavily used if a student reported use 4 or more times in the previous 7 days (Figure 1, Table 1). Most respondents use Google on a daily basis (154/202, 76.2%; 95% CI 69.8%-81.9%), and very few reported using the search engine on fewer than 4 of the prior 7 days (13/202, 6.4%; 95% CI 3.5%-10.8%). Wikipedia, UpToDate (a subscription-only, evidence-based summary source), and personal/provided notes were also heavily used. In contrast, few respondents (28/199, 14.1%; 95% CI, 9.6%-19.7%) had used bibliographic databases on at least 4 days, and just over one-third (68/199, 34.2%; 95% CI, 27.6%-41.2%) had not used such sources at all in the previous week.

Comparison of preclinical- and clinical-level respondents revealed two prominent differences: clinical-level respondents reported less frequent use of bibliographic databases (*P*<.001) and more frequent use of UpToDate (*P*<.001) (Table 1).

**Figure 2:**
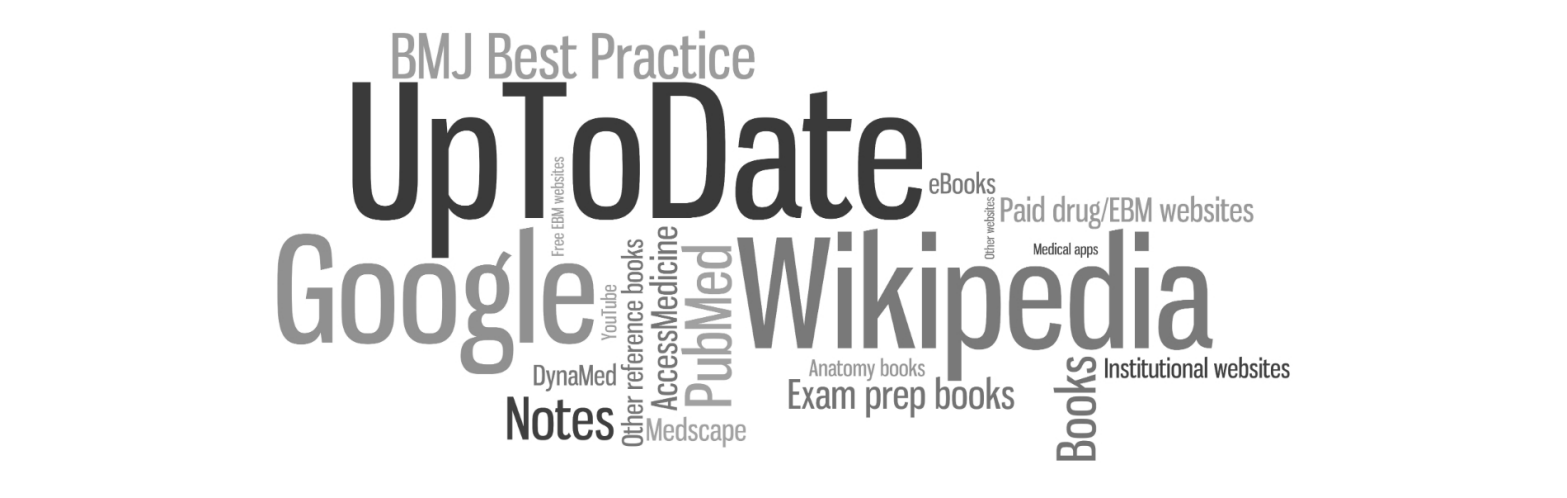
Student-identified top resources from the previous 7 days.

Of note, these results were somewhat different from respondents’ self-generated lists of most used resources. Here, UpToDate was the most-listed single reference, while Google and Wikipedia were the next most commonly cited resources (Figure 2).

**Table 1:** Preclinical and clinical student use of resources during the previous 7 days.

Source			Responses	Never n (%)	Once n (%)	2-3 times n (%)	4-6 times n (%)	Daily n (%)
**Google**			202	0 (0)	1 (0.5)	12 (5.9)	35 (17.3)	154 (76.2)
	Preclinical		102	0 (0)	0 (0)	8 (7.8)	19 (18.6)	75 (73.5)
	Clinical		99	0 (0)	1 (1.0)	4 (4.0)	16 (16.2)	78 (78.8)
**Wikipedia**			201	6 (3.0)	19 (9.4)	48 (23.9)	45 (22.4)	83 (41.3)
	Preclinical		101	4 (4.0)	8 (7.9)	25 (24.8)	25 (24.8)	39 (38.6)
	Clinical		99	2 (2.0)	11 (11.1)	23 (23.2)	20 (20.2)	43 (43.4)
**Notes**			197	19 (9.6)	20 (10.2)	54 (27.4)	39 (19.8)	65 (33.0)
	Preclinical		100	9 (9.0)	7 (7.0)	34 (34.0)	18 (18.0)	32 (32.0)
	Clinical		96	10 (10.4)	12 (12.5)	20 (20.8)	21 (21.9)	33 (34.4)
**UpToDate**			201	35 (17.4)	13 (6.5)	31 (15.4)	65 (32.3)	57 (28.4)
	Preclinical		101	30 (29.7)	7 (6.9)	18 (17.8)	37 (36.6)	9 (8.9)
	Clinical		99	5 (5.0)	6 (6.1)	13 (13.1)	27 (27.3)	48 (48.5)
**Bibliographic databases**			199	68 (34.2)	44 (22.1)	59 (29.6)	20 (10.0)	8 (4.0)
		Preclinical	100	19 (19.0)	18 (18.0)	40 (40.0)	16 (16.0)	7 (7.0)
		Clinical	98	49 (50.0)	26 (26.5)	18 (18.4)	4 (4.1)	1 (1.0)

### Values and Reasons for Using Sources

**Figure 3:**
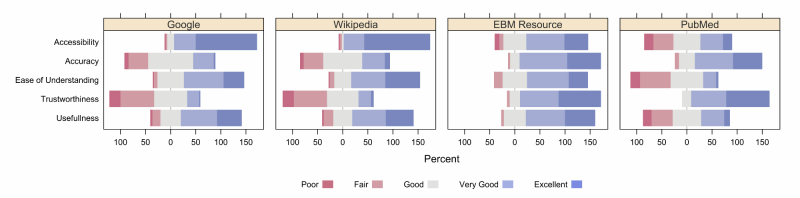
Student evaluations of specified resources.

Whereas students valued the general purpose resources Google and Wikipedia highly for their accessibility, understandability, and usefulness, they gave PubMed and other bibliographic databases stronger ratings for accuracy and trustworthiness. As a representative EBM summary, UpToDate appeared to bridge this gap: it received high ratings across values although many students noted elsewhere that access to this resource was limited by cost and lack of an institutional subscription (Figure 3).

Students identified key factors in their impressions of a source’s trustworthiness: recognition of factual errors in a reference and reputation among mentors and peers, as well as being specifically counseled to use or avoid a reference.

### Self-Identified Needs and Abilities

Students offered valuable insights in their responses to open-ended questions, and several themes emerged. Many spoke of being overwhelmed by the number of available and/or suggested resources as well as by the density of information in these sources; respondents repeatedly reported a need for more basic information and for managing information/source overload. They requested faculty- and/or peer-generated resource recommendations, although some noted that current lists added to a sense of overload. Perhaps in response to this challenge, several requested increased and longitudinally integrated instruction on searching for information and often highlighted a desire to learn how to approach nontraditional, or general purpose, Internet sources. Finally, resource accessibility presented an additional, practical barrier to finding useful information. Respondents commonly identified a need for freer access, including at the point of care; they cited lack of universal Internet access, inconvenience of multiple sign-ins, and subscription-based access as barriers.

## Discussion

### Dalhousie University Medical School Curriculum

Our survey assessed patterns of information resource use among students in the undergraduate MD program at Dalhousie University. Students in the program must have an undergraduate degree; some have additional study or work experience. The school has two campuses (Halifax, Nova Scotia, and Saint John, New Brunswick); the sites share a uniform curriculum, and many lectures are videoconferenced. All students have remote access to Dalhousie University library electronic resources throughout their training.

The medical school follows a 4-year curriculum. Two classroom-based preclerkship years aim to provide a foundation of knowledge and basic skills. A two-year clerkship follows, spent mostly in clinical settings.

A concentrated series of lectures on EBM are delivered at the beginning of year one. On a recent syllabus, seven hours during that unit were devoted to aspects of EBM, including five hours on question formulation and searching for evidence. Lecture notes refer to a standard hierarchy of preappraised evidence and discuss how and when to use various resources [Robin Parker, Sources of evidence, 10 September 2012, Dalhousie University, Canada].

Information management and EBM continue to receive attention at various points after this introductory unit. Weekly sessions during the first two years address evidence search and appraisal and other topics (ethics, law, population health, and professionalism). The formal curriculum is more limited during the clinical years, and much of the planned teaching time addresses clinical skills; training objectives continue to refer to critical use and appraisal of evidence.

Students have access to an array of resources and resource guides. They receive reference recommendations for each unit, and notes and links are posted on general and course-specific websites. The library website houses subject guides for each course and clinical rotation, including guides to search strategies for PubMed and Cochrane Library, a subscription-based set of medical and healthcare databases.

### Principal Findings

#### Behavior Diverges From Formal Instruction

In this survey, most students recalled having received formal instruction on health information searches during their training, most of which concerned traditional information sources such as PubMed or other bibliographic databases. Although students commonly reported being discouraged from using certain resources and seldom recalled instruction in the use of general purpose search resources (such as Google), they reported Google as the most frequently used in their actual practice. The contrast between self-reported formal instruction and practice may have several explanations. Medical education takes place on multiple levels: while the formal curriculum transmits intentional and explicit instruction, informal and hidden curricula operate through experience, interactions, and role modeling [28]. A substantial body of literature avers the importance of these latter curricula on the emerging professional and ethical identities of the students. Our respondents likewise rated reputation among peers and mentors as an important factor in their valuation of information sources. Informal curricula may well influence learning behaviors as much as they do ethical ones. If so, given the high rates of general purpose and preappraised resource use among medical residents and qualified physicians [9,11,12]—students’ chief mentors during training—it is not surprising that these students should so frequently use such resources. Moreover, increased informal, experiential interaction with these mentors during the clinical training years would be expected to result in increased use of role-modeled resources.

#### Changing Patterns of Resource Use

Students in their clinical years used Google heavily but reported increased use of UpToDate and less reference to bibliographic databases compared to their preclinical peers. With the shift to a clinical setting, students’ information needs change: they require more patient-oriented information, whereas preclinical students must accumulate basic knowledge of physiology and disease. As well, the real-world, clinical setting demands increased search efficiency, often making review of individual studies impractical [4]. Such needs may have influenced the students in this study and have spurred an industry of preappraised, summarized information sources (including UpToDate). Although criticized for variable design and timeliness [29,30], preappraised summaries have proven superior to primary literature searches in some cases [31,32] and are commonly considered an important part of evidence-based practice [33].

Access to EBM summaries may have been a contributing factor to the seniority-related variation in resource use. Anecdotally, several students told us that they purchase or share subscriptions to resources, including UpToDate, upon entering clerkship. While our university does not hold a license for UpToDate—the EBM summary most frequently named by students—some clinical settings do.

Meanwhile, the fact that students less commonly named evidence summaries to which our university *does* offer access is significant. It may reflect the strength of peer and mentor influences. It could also speak to the problem of information resource overload. Excess information may lead to errors of omission in the clinical context [34,35]: with such a large variety and volume of resources, information may be lost. One medical librarian’s observation that “students may not have an accurate picture of the access they’re entitled to” [personal communication by Kathleen Gadd, 9 March 2015] lends support to this possibility.

#### Resource Selection: A Balance of Needs

The values respondents attributed to various information sources identify ease of understanding and accessibility as common features among the more heavily used resources. Meanwhile, their comments highlight challenges navigating a surplus of information and managing source accessibility. Their practice appears to reflect a trade-off between accessibility/digestibility and accuracy: students appear to believe the balance of benefit lies with summary sources, whether medical or general purpose.

Such calculations constitute a satisficing approach to searching and source selection. Faced with practical limits on obtaining and analyzing a large volume of relevant data, individuals select what they perceive to be a good enough option [36-38]—for example, selecting Google or UpToDate to locate a piece of needed information instead of conducting a thorough literature review. The concern, of course, is whether students are equipped to know and choose what is actually good enough. Can they appraise the source and the information,adequately to determine whether it meets minimum standards? An optimistic view would hold that this is the case. On the other hand, given the lack of instruction on use and appraisal of their most-used sources, we might question the bases for their strategies and consider the risks of uncritical information seeking.

#### Student Self-Assessment

A strong majority of respondents rated their information seeking skills as good or better—this despite reporting information search practices that diverged from the formal instruction they had received. Perhaps this is because they achieve good results with their current search practices. It is worth noting, however, the limits inherent to self-assessment. Individual self-rating bears little relation to actual competence, and most students overestimate their performance; this is demonstrable among medical students, among others [39-41]. Individuals who believe they have adequate skills are unlikely to seek remediation, whether or not they are truly competent. Furthermore, the students in this survey reported feeling overwhelmed by the information landscape; we know medical professionals consistently choose CME activities that address interests and skills rather than weaknesses and can surmise that medical student behavior is likely similar. It seems unlikely that these students will independently seek out ways to improve their skills, now or possibly even into their careers. If there is a better approach to seeking information, medical educators must offer active guidance.

### Study Limitations

Our study has limitations. We conducted the survey at a single medical school. The response rate was lower than desired. Our response rate appears comparable, however, to that achieved by previous online surveys addressing technology and information management in medicine [20-22,42].

Our survey relied on self-reported behaviors, which are known to be subject to social desirability bias. External observation of behavior, however, is not feasible for the current inquiry. A daily diary approach might have offered greater accuracy but at a further cost to the response rate. We asked respondents to report practices from the preceding 7 days as a strategy to obtain more accurate self-reported data than a more general inquiry. Despite this, the difference seen between the frequencies of use reported for different resources and respondents' self-generated lists of most-used resources suggested the possibility of bias in some questions. We would, however, have expected social desirability bias to skew results away from non-traditional, general-purpose sources—instead we saw quite a dramatic favoring of such resources over bibliographic databases.

Our survey obtained a cross-sectional assessment, with reference to behavior during the preceding seven days. It is possible that student behavior differed from usual practice during the week prior to the survey, rendering nonrepresentative results. In an effort to limit this possibility, we discussed survey timing with the Dalhousie Undergraduate Medical Education Curriculum Committee and student leaders and selected survey dates that fell during the routine session, avoiding examination or recess periods.

### Conclusions

No tool is optimal for every purpose. Students need to gain skills and familiarity assessing primary literature, but they also must learn to find useful, practical information efficiently. They need to recognize what tool will best serve a given purpose—whether this is getting an overview of a common condition or physiologic process or assessing effectiveness of alternate medical therapies—and to appreciate the limitations inherent to that tool. Thus, whereas current formal instruction on information searching neither reflects nor appears to alter,student behavior, consideration should be given to instruction on information management and appraisal in general. Medical education that includes use and appraisal of primary literature, summary sources, and even general purpose resources might more effectively equip students in their pursuit of lifelong learning.

## Appendix

Multimedia Appendix 1 Survey instrument.

## Abbreviations

EBM: evidence-based medicine
