# Supplementary material for: Information-Seeking Behaviors of Medical Students: A Cross-Sectional Web-Based Survey
Source: JMIR Med Educ. 2015 Jun 29;1(1):e4. doi: 10.2196/mededu.4267 (PMC5041342; doi:10.2196/mededu.4267)
Supplement: Multimedia Appendix 1 [file mededu_v1i1e4_app1.pdf]

Multimedia Appendix 1: Survey instrument

Survey of Medical Students' Information and Learning Behaviour

Thank you for agreeing to participate in this survey!

Medical school challenges students to seek out and learn new information all the time, whether in the classroom or on clinical rotations. We hope to gain a better understanding of medical students' strategies for gathering information, so that we can help to develop curricula that better meet your needs. Click here for more information. *(link to page with further information on the project and participation, including confidentiality and researchers' contact information)*

This survey will take about ten minutes.

To start the survey, please select the "Start" button below. To exit without participating, select "Decline."

- ○ Start
- ○ Decline

Question 1 of 20
What year were you born?

19 ☐
YY

Question 2 of 20
What is your gender?

- ○ Male
- ○ Female
- ○ Prefer not to answer

Question 3 of 20
What is your current year of medical school?

- ○ 1st year
- ○ 2nd year
- ○ 3rd year
- ○ 4th year

Question 4 of 20
As part of your medical education, have you received formal instruction on how to search for health information?

- ○ Yes
- ○ No
- ○ Not sure/don't remember

Question 5 of 20
Have you been discouraged from using any information resources (e.g., Google, Wikipedia, blogs, YouTube, Facebook)?

- ○ Yes
- ○ No
- ○ Not sure/don't remember

Question 6 of 20
As part of your medical education, have you received formal instruction on how to use the following resources to find health information?

	Yes	No	Not sure/don't remember
Google	○	○	○
Other search engines	○	○	○
Pubmed or other bibliographic databases	○	○	○
Wikipedia	○	○	○
Other social media (e.g., blogs, YouTube, Facebook)	○	○	○

Question 7 of 20
How do you rate your skills in using the Internet for health information?

- ○ Excellent
- ○ Very good
- ○ Good
- ○ Fair
- ○ Poor

Question 8 of 20
Have you posted health information for the general public online?

- ○ Yes
- ○ No

Question 9 of 20
Have you contributed to a peer-reviewed publication?

- ○ Yes
- ○ No

Question 10 of 20
In the past 7 days, how often did you use the following <u>types</u> of resources for health information? If, for example, you used Google and followed a link to a Wikipedia article, count each resource separately.

	Never	Once	2 to 3 times	4 to 6 times	Daily
Bibliographic databases (e.g., PubMed)	○	○	○	○	○
Book	○	○	○	○	○
E-book	○	○	○	○	○
Google	○	○	○	○	○
Other search engines	○	○	○	○	○
Notes (personal or shared/provided)	○	○	○	○	○
UpToDate	○	○	○	○	○
Websites by government or medical organizations	○	○	○	○	○
Wikipedia	○	○	○	○	○
Other social media websites (e.g., blogs, YouTube, Facebook)	○	○	○	○	○
Other (please describe) ________	○	○	○	○	○

Question 11 of 20
In the past 7 days, how many individual reference sources have you used for health information? Please enter a number below. *An individual book, file, search engine, database, website, or app is a single reference source. If you used two different websites last week, you used two reference sources. If you used one website several times, you used one reference source.*

[ ] # of sources

Question 12 of 20
Please list the five reference sources you used most often for health information in the past 7 days.

Most used reference source	
2nd most used reference source	
3rd most used reference source	
4th most used reference source	
5th most used reference source	

Question 13 of 20
How well does Google meet your health information needs, according to the following features?

	N/A	Poor	Fair	Good	Very Good	Excellent
Accessibility	○	○	○	○	○	○
Accuracy	○	○	○	○	○	○
Ease of understanding	○	○	○	○	○	○
Trustworthiness	○	○	○	○	○	○
Usefulness	○	○	○	○	○	○

Question 14 of 20
How well does Wikipedia meet your health information needs, according to the following features?

	N/A	Poor	Fair	Good	Very Good	Excellent
Accessibility	O	O	O	O	O	O
Accuracy	O	O	O	O	O	O
Ease of understanding	O	O	O	O	O	O
Trustworthiness	O	O	O	O	O	O
Usefulness	O	O	O	O	O	O

Question 15 of 20
How well does PubMed (or your preferred bibliographic database—e.g., Ovid, CINAHL) meet your health information needs, according to the following features?

	N/A	Poor	Fair	Good	Very Good	Excellent
Accessibility	O	O	O	O	O	O
Accuracy	O	O	O	O	O	O
Ease of understanding	O	O	O	O	O	O
Trustworthiness	O	O	O	O	O	O
Usefulness	O	O	O	O	O	O

Question 16 of 20
How well does UpToDate (or your preferred EBM resource—e.g., BMJ Best Practice, Dynamed) meet your health information needs, according to the following features?

	N/A	Poor	Fair	Good	Very Good	Excellent
Accessibility	O	O	O	O	O	O
Accuracy	O	O	O	O	O	O
Ease of understanding	O	O	O	O	O	O
Trustworthiness	O	O	O	O	O	O
Usefulness	O	O	O	O	O	O

Question 17 of 20

How well does [ ] meet your health information needs, according to the following features? Please enter the name of your most-used resource, excluding those already discussed.

	N/A	Poor	Fair	Good	Very Good	Excellent
Accessibility	○	○	○	○	○	○
Accuracy	○	○	○	○	○	○
Ease of understanding	○	○	○	○	○	○
Trustworthiness	○	○	○	○	○	○
Usefulness	○	○	○	○	○	○

Question 18 of 20

How important are the following factors to your opinion of a reference source's trustworthiness?

	Not important	Slightly important	Somewhat important	Very important	Extremely important
Author credentials	○	○	○	○	○
Funding source	○	○	○	○	○
Reputation with peers	○	○	○	○	○
Reputation with mentors	○	○	○	○	○
Factual errors identified	○	○	○	○	○
Advised for/against use by instructor(s)	○	○	○	○	○

Question 19 of 20
What would help you improve your use of Internet sources for health information?

Question 20 of 20
We would appreciate any further comments you may have about using Internet sources for health information.

Thank you for your participation in this survey. We value your contribution to our understanding of medical students' information and learning practices.

The survey is now complete and your responses have been saved. It is safe to close or redirect your browser window.
